# Hemoprotein Catalyzed Oxygenations: P450s, UPOs, and Progress toward Scalable Reactions

**DOI:** 10.1021/jacsau.1c00251

**Published:** 2021-07-26

**Authors:** Gideon Grogan

**Affiliations:** Department of Chemistry, University of York, Heslington, York YO10 5DD, United Kingdom

**Keywords:** Cytochromes P450, Unspecific Peroxygenase, Biocatalysis, Oxygenation, Biotransformation

## Abstract

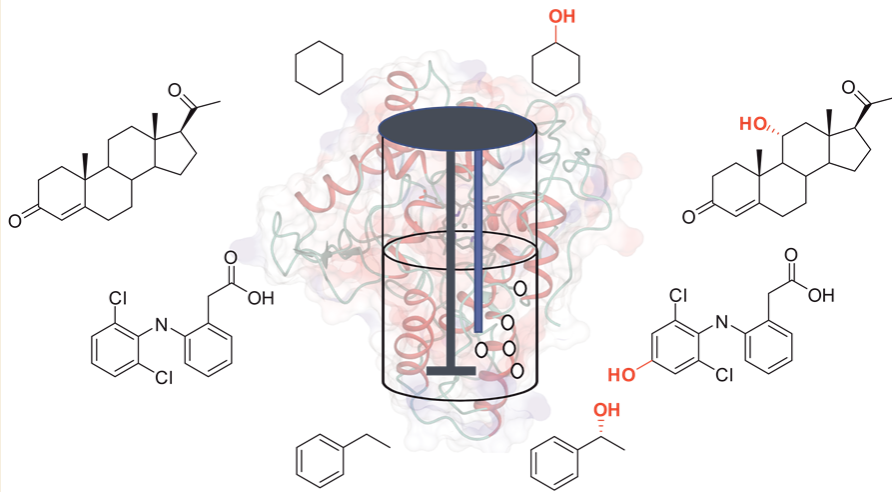

The selective oxygenation of nonactivated carbon atoms is an ongoing synthetic challenge, and biocatalysts, particularly hemoprotein oxygenases, continue to be investigated for their potential, given both their sustainable chemistry credentials and also their superior selectivity. However, issues of stability, activity, and complex reaction requirements often render these biocatalytic oxygenations problematic with respect to scalable industrial processes. A continuing focus on Cytochromes P450 (P450s), which require a reduced nicotinamide cofactor and redox protein partners for electron transport, has now led to better catalysts and processes with a greater understanding of process requirements and limitations for both in vitro and whole-cell systems. However, the discovery and development of unspecific peroxygenases (UPOs) has also recently provided valuable complementary technology to P450-catalyzed reactions. UPOs need only hydrogen peroxide to effect oxygenations but are hampered by their sensitivity to peroxide and also by limited selectivity. In this Perspective, we survey recent developments in the engineering of proteins, cells, and processes for oxygenations by these two groups of hemoproteins and evaluate their potential and relative merits for scalable reactions.

## Introduction

**Scheme 1:**
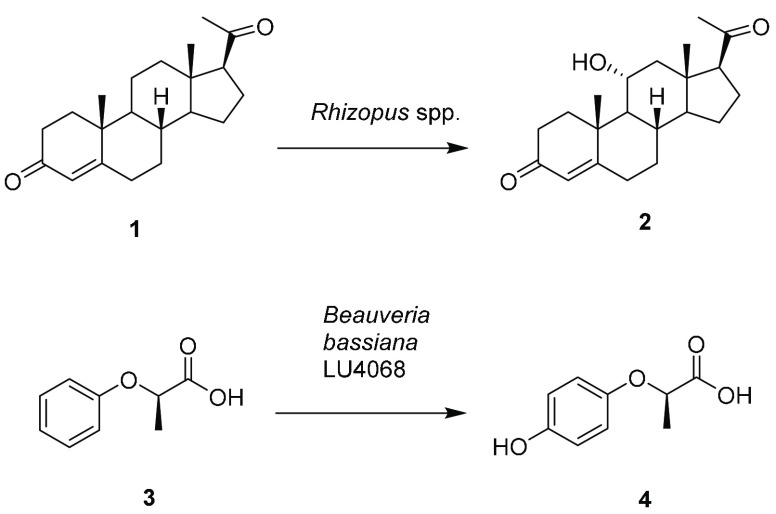
Examples of Scalable Hydroxylations by Whole Cells of Filamentous Fungi

The selective oxygenation of nonactivated carbon centers remains a significant challenge in organic chemistry and has been addressed over several decades using either microorganisms or enzymes.^1,2^ Biocatalyzed oxygenations have advantages over chemical reagents that accomplish similar, if less selective, transformations: They occur at ambient temperatures and pH and do not utilize harmful metals such as chromium. However, it is arguably their selectivity that makes them so highly prized. The observation that the fungus *Rhizopus* spp. hydroxylated progesterone **1** to 11α-hydroxyprogesterone **2** (Scheme 1) in a regio- and stereoselective manner was a revelation as far back as 1952,^3^ and the further development of whole-cell fungal biocatalysts for preparative scale oxygenations held, and continues to hold, much promise.^4^

In another notable example, Hauer and colleagues used a chemically mutated strain of the fungus *Beauveria bassiana* to hydroxylate the pesticide precursor (*R*)-phenyl propionic acid **3** to hydroxyphenylpropionic acid **4** (HPOPS) at the 100 m^3^ scale with a productivity of 7 g L^–1^ d^–1^ (Scheme 1).^5^ These and other advances created an expectation that biocatalytic oxygenation would be a breakthrough technology for the selective synthesis of pharmaceuticals, agrochemicals, drug metabolites, and also bulk chemicals. Enthusiasm would have been increased by the revolution in molecular biology and genomics in subsequent years, which revealed a vast number of gene sequences encoding “hydroxylase” enzymes of many types and also the genetic engineering tools to express them in heterologous hosts and to improve their catalytic abilities using protein engineering. Hemoproteins have, for the most part, been the enzymes of choice for selective oxygenations, and indeed, the cytochromes P450 (P450s),^6,7^ a large family of hemoproteins, are responsible for catalyzing the reactions shown in Scheme 1. The diversity of chemical reactions catalyzed by hemoproteins means that, in theory, a wide range of biotransformations to valuable products should be achievable, in modes similar to those achieved in the 1950s with steroids, if techniques could be developed to express these enzymes in generally regarded-as-safe (GRAS), easy-to-grow host organisms. However, despite the number of genes, and the tools available to clone, express, and mutate them, the number of scalable industrial biocatalytic oxygenations, outside those that exploit native strains or those altered by “Classical Strain Improvement” (CSI, using mutagens), remains low. In this Perspective, the recent (since 2014) literature on progress toward scalable biooxygenations using hemoprotein oxygenases is surveyed. Advances in protein engineering and process technology for exploiting recombinant P450s in vitro and in whole cells are considered. These developments will then be contrasted with parallel work on another class of hemoproteins, the unspecific peroxygenases (UPOs),^8,9^ which have emerged as a complementary set of enzymes with the potential for scalable oxygenation reactions.

## P450s and UPOs: A Brief Introduction

**Scheme 2:**
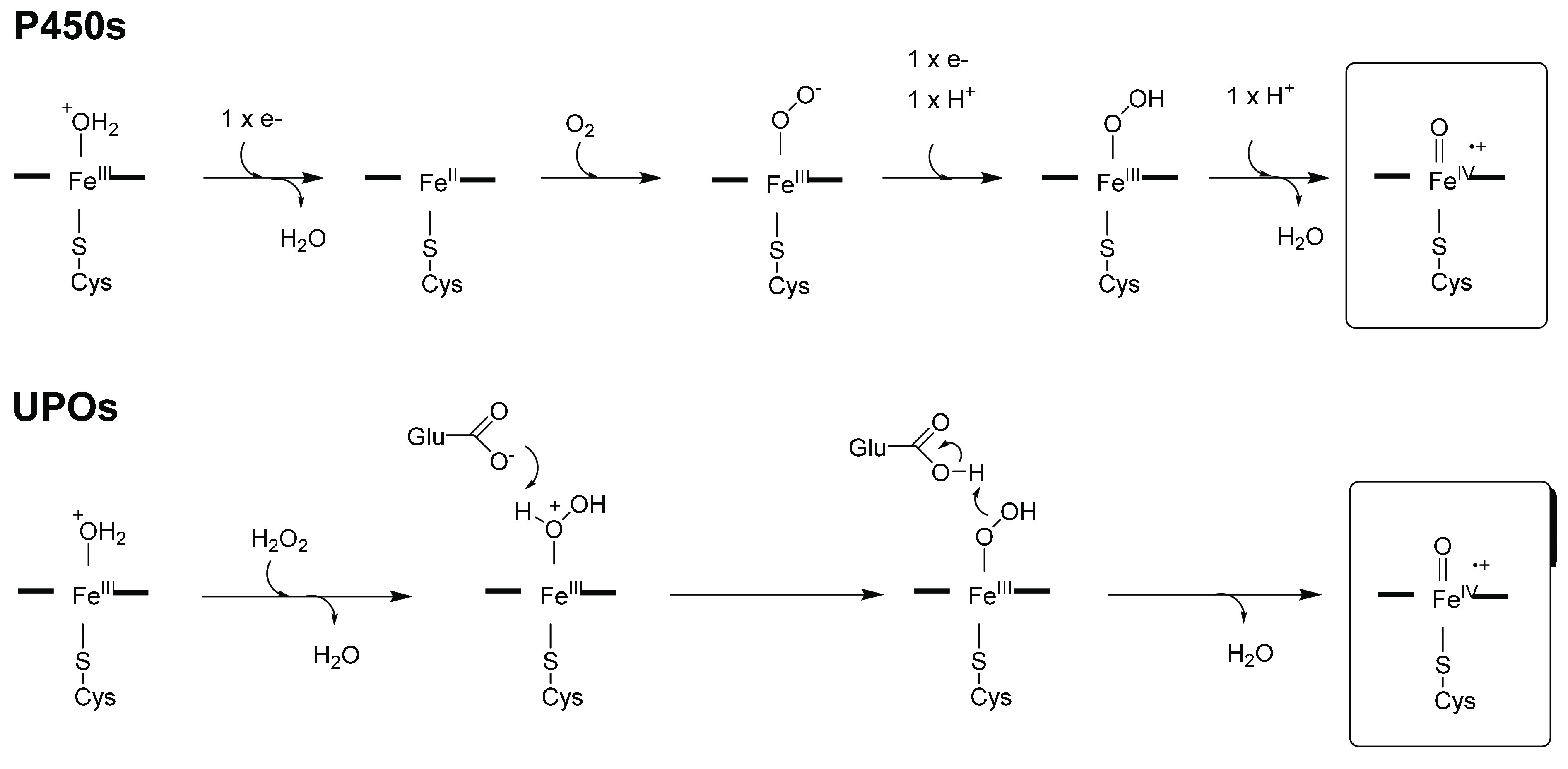
Formation of Compound I (Boxed) in P450s and UPOs

Before considering recent developments, it is useful to briefly contrast the biochemical characteristics of P450s and UPOs. In both enzymes, oxygenation of a carbon atom is accomplished by an iron(IV) oxo species (“Compound I”, Scheme 2) formed at the heme iron within the active site. The mechanism of formation of this species differs between the two enzyme classes and there are fundamental differences in the active site structure that enable Compound I to be formed in each case. In P450s, following substrate binding, and a one electron reduction of Fe^3+^ to Fe^2+^, the iron reacts with dioxygen, and the further delivery of another single electron enables the cleavage of the dioxygen bond via an iron peroxo complex, yielding water as a byproduct, to give Compound I (Scheme 2).^7,10^

**Figure 1:**
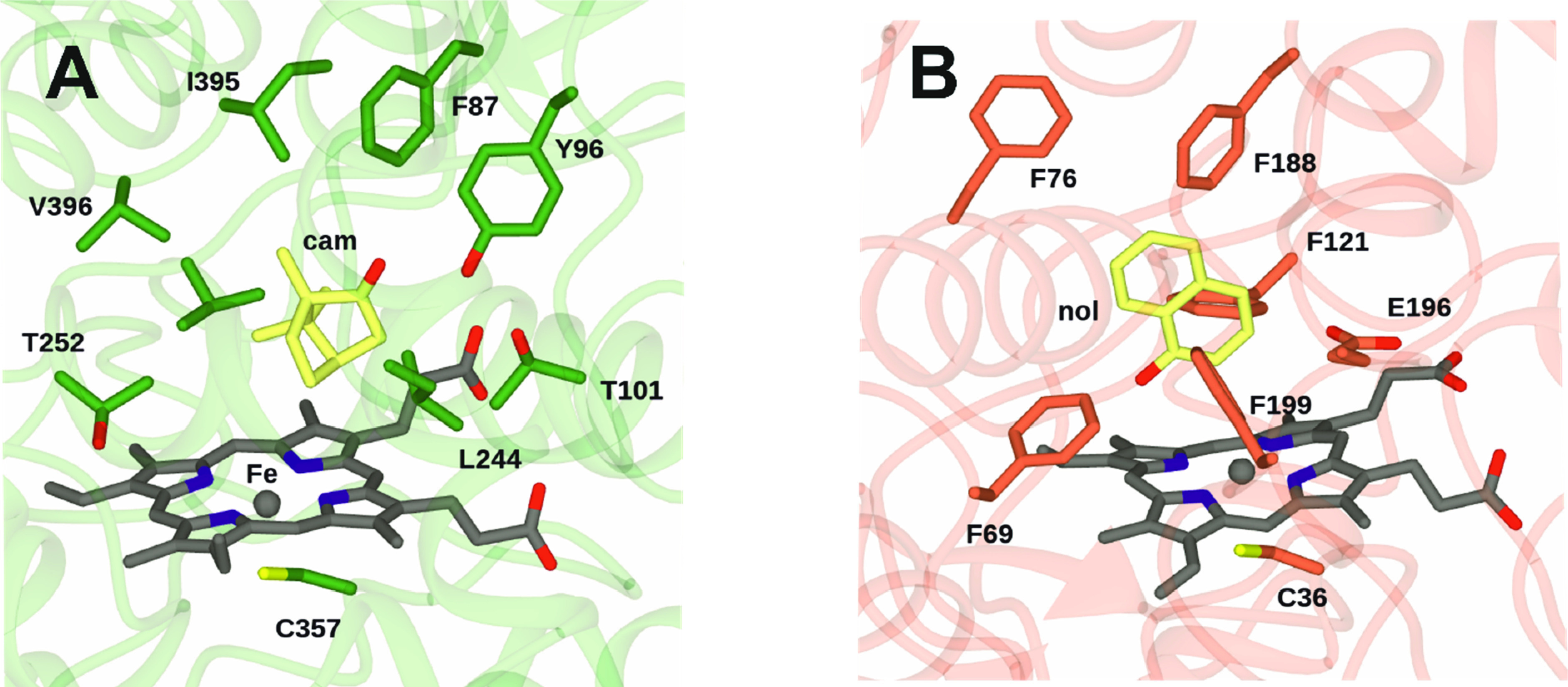
(A) Active site of P450cam in complex with camphor (cam) (PDB 1CPP). (B) Active site of *Agrocybe aegerita* (*Aae* UPO) complexed with 1-naphthol (nol) (PDB 6EKY). One ligand from these coordinates has been selected for clarity.

In UPOs, reaction of the iron with hydrogen peroxide (H_2_O_2_) gives a ferric peroxo complex directly and this also cleaves to give Compound I (Scheme 2).^8,11,12^ In both P450s and UPOs, the heme iron is ligated at the axial position by a cysteine residue. However, the mechanism of O–O bond cleavage for Compound I formation is assisted by different residues in the active site of each enzyme (Figure 1): In the well-studied P450cam, this is thought to be catalyzed by a threonine residue T252 (Figure 1A);^13^ and in UPOs it is thought to be catalyzed by a glutamate E196, situated above the heme (Scheme 2; Figure 1B).^14^

In P450s, the formation of Compound I depends on the delivery of electrons to the heme by electron transport proteins, the identity and nature of which are diverse.^15^ In the most common systems, electrons are delivered from a nicotinamide cofactor NAD(P)H, via either a flavin-containing ferredoxin reductase (FdR) and an iron–sulfur containing ferredoxin (Fd) (“Class I”,^15^ found in bacteria) or a flavin-containing cytochrome P450 reductase (CPR) alone (“Class 2”; in fungi, plants, and mammals). The requirement for both expensive nicotinamide cofactors and these electron-transfer proteins renders the application of recombinant P450s in vitro somewhat complex, as more than one gene must be expressed, and there is a lack of efficiency in electron transfer, giving rise to what is termed “uncoupling”. In some cases, the electron transport proteins and hemoprotein can be naturally fused within one polypeptide, simplifying the problems of expression and reconstitution *in vitro*. The most famous example is cytochrome P450BM3 (CYP101A2) from *Bacillus megaterium*,^16^ which is probably the most studied and engineered P450 for process applications for this reason. Some P450s are also able to operate in a “peroxygenase” mode, in which the addition of H_2_O_2_ to the heme domain in the absence of electron transfer proteins leads to the formation of Compound I at the heme, and enables hydroxylation to occur.^17^ Indeed some P450s are known to operate naturally in peroxygenase mode,^18,19^ and have been the focus of considerable recent interest.

The H_2_O_2_-dependent generation of Compound I is also employed by UPOs, which, consequently, are not dependent for activity on NAD(P)H or electron transfer proteins. UPOs are secreted enzymes expressed in Nature from fungi and are thought to form part of the biocatalytic arsenal deployed for the degradation of complex biomolecules such as lignin.^8,9^ UPOs, which were first identified by Hofrichter et al. in 2004,^20^ were initially thought to be chloroperoxidases, another hemoprotein that deploys Compound I for oxygenations, although in that case the oxidation of halide ions to form X^+^ ions that are employed in the electrophilic halogenation of largely aromatic substrates. However, research on their structure and biochemistry has revealed the significantly greater oxidative power of UPOs, in that they are also capable of the hydroxylation of a wide variety of organic compounds. However, as with P450s, there are issues: UPOs display a sensitivity to the H_2_O_2_ that they employ to make Compound I. There is also a comparative lack of selectivity that leads to multiple products with substrates having comparably susceptible carbon centers. UPOs, being eukaryotic enzymes, are also glycosylated, and most examples appear to require glycosylation for full activity, so heterologous expression in most cases has been restricted to yeasts such as *Saccharomyces cerevisiae* and *Pichia pastoris*.

The apparent advantages and disadvantages of P450s and UPOs as oxygenase catalysts with the potential for scalable reactions are listed in Table 1. In 2015, Lundemo and Woodley summarized the challenges of using P450 systems at scale,^21^ some of which may apply to UPOs. In their summary, “biological” considerations included biocatalyst stability, to maximize the benefits of the best turnover numbers seen in P450 reactions, robust heterologous hosts for whole-cell reactions, and maximization of coupling within the electron transfer system. “Process” factors to be considered included ease of product recovery and substrate and product characteristics and how these inform the choice of system for scale-up.

**Table 1:** Overview of Catalytic Characteristics of P450s and UPOs

characteristic	P450s	UPOs
require electron transport proteins or domains	yes	no
requirement for nicotinamide cofactors and cofactor recycling	yes	no
uncoupling reactions	yes	no
use H_2_O_2_	sometimes	yes
sensitivity to H_2_O_2_	yes	yes
expression in *E. coli*	yes	some examples
expression in yeasts	yes	yes
whole-cell systems for hydroxylation	yes	no

In this Perspective, we will compare and contrast advances that have addressed the limitations of each enzyme type and that look forward to their wider application in the future. We show that some of the issues have been addressed directly for P450 catalysis using both in vitro and whole-cell systems but that sometimes reaction outcomes may be better realized through the application of the alternative UPOs.

## Cytochromes P450

### Enzyme Engineering for In Vitro Applications

The generation of useful activity is the first step in developing a biocatalytic process, and new activities of P450s, both natural^22^ and non-natural,^23^ continue to be discovered. The group of Arnold has, for example, shown that P450s can be engineered for cyclopropanation^24^ and intermolecular amination^25^ reactions and the hydroxylation of silanes.^26^ However, the focus of many projects has been the in vitro evolution of enzymes, predominantly P450BM3, which possess the qualities of high native activity and fusion of reductase and hemoprotein domains, for selective oxygenation reactions.

### Hydroxylation of Steroids by P450BM3 Mutants

**Scheme 3:**
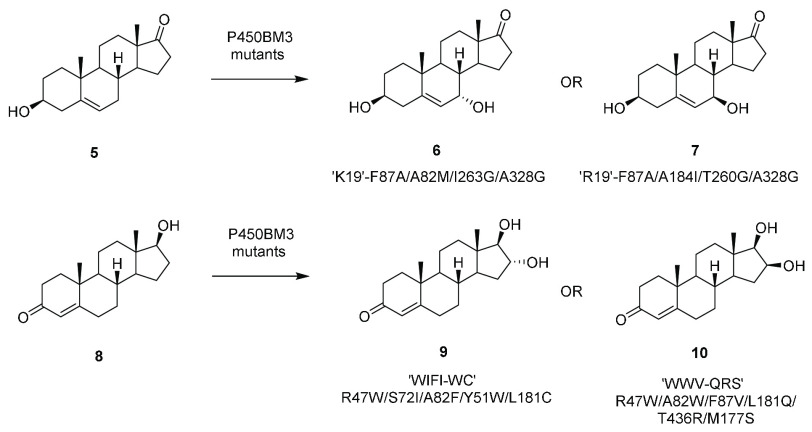
Regioselective Hydroxylation of Steroids by P450BM3 Mutants

Given the significance of steroids to the pharmaceutical industry and the history of P450s in steroid transformations, they remain a focus for P450 evolution. Libraries of P450BM3 variants were assessed for the selective hydroxylation of dehydroepiandrosterone (DHEA) **5**, testosterone (TST) **8**, and androstenedione (AD) (Scheme 3).^27^

A comparison of P450BM3 and the steroid C19-demethylase CYP19A1 suggested glycine scanning as an effective approach to the discovery of mutants with improved regioselectivity. For example, mutant “K19” F87A/A82M/I263G/A328G was 93% selective for generating 7-α-hydroxy DHEA **6**, whereas “R19” F87A/A184I/T260G/A328G was 97% selective for 7-β-hydroxy DHEA **7** (where K19 and R19 represent starting points of already constructed mutants). Mutations at positions 260 and 263 were thought to distort the I-helix in the heme domain of P450BM3, creating space for complementary steroid binding modes. These experiments were performed at the 2 mM [substrate] scale in 200 mL and employed cofactor recycling. Li and co-workers started with the triple variant F87G/A328G/A330W and targeted 15 further sites at or near the active site for mutagenesis.^28^ Variant “LG-23”, with 14 mutations, was 83% selective for the production of 7β- hydroxytestosterone. Reactions were scaled up to 1 mM [substrate] in 50 mL using freeze-thawed cells, giving products in up to 82% yield. 7β-Selectivity was also observed for LG-23 with four other steroids including nandrolone. The same group used an in vitro evolution approach based on mutability landscapes, structure, and modeling to create variants that targeted the 16-position of testosterone.^29^ Mutant WIFI-WC (R47W/S72I/A82F/S72I-Y51W/L181C) was 96% selective for the production of 16α-testosterone **9**, whereas WWV-QRS (R47W/A82W/F87V-L181Q/T436R/M177S) was 92% selective for the 16β-isomer **10**. Scaled-up reactions using freeze-thawed cells at 1 mM [substrate] in 100 mL gave 23.3 and 7.7 mg of products **9** and **10** with yields of 77% and 27%, respectively.

### Aromatic Hydroxylations by P450BM3 Mutants

Reetz and co-workers engineered P450BM3 mutants for the oxidation of benzene through phenol to hydroquinone.^30^ Variant A82F/A328F converted phenol to hydroquinone, with a turnover rate of 590 min^–1^, but also converted benzene to hydroquinone with 90% conversion. The mutant was employed in whole cells at the 20 mL scale in 10 mM benzene, giving the product in 55% yield. The conversion of benzene to phenol was also accomplished by Shoji and co-workers using the “decoy” molecule 3-CPPA-Pip-Phe to stimulate the hydroxylation of the substrate.^31^ Wild-type (wt) P450BM3 in whole cells of *Escherichia coli* converted 20 mM benzene to phenol at the 1 mL scale, with a total turnover number (TTN) of 54 500, with no conversion to hydroquinone.

**Scheme 4:**
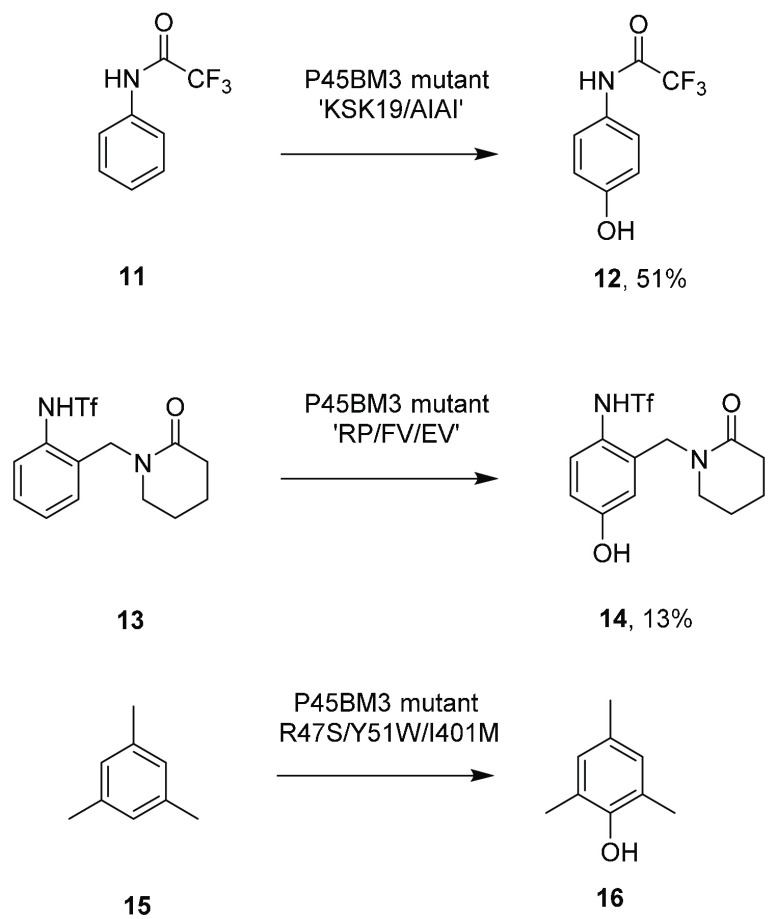
Hydroxylation of Aromatic Compounds by P450BM3 Mutants

A range of anilides was transformed by P450BM3 variants to phenols in studies by O’Hanlon et al.^32^ Trifluoroacetanilide **11** was transformed to phenol **12** with 100% conversion by enzyme “KSK19/AIAI” in vitro at the 0.079 mol scale in 30 mL, giving a yield of 51% (Scheme 4).

The herbicidal *N*-sulfonylanilide **13** was hydroxylated to **14** by the P450BM3 mutant “RP/FV/EV” at the 0.9 mmol scale in 34 mL in 13% yield. Dennig and co-workers showed that variant “M3” (R47S/Y51W/A330F/I401M) converted mesitylene **15** (1,3,5-trimethylbenzene) to **16** with 89% selectivity. The reaction was performed at the 50 mL scale with 4 mM [substrate] using cell-free extract (CFE) and represented a 230-fold improvement in activity over wt P450BM3.^33^

**Scheme 5:**
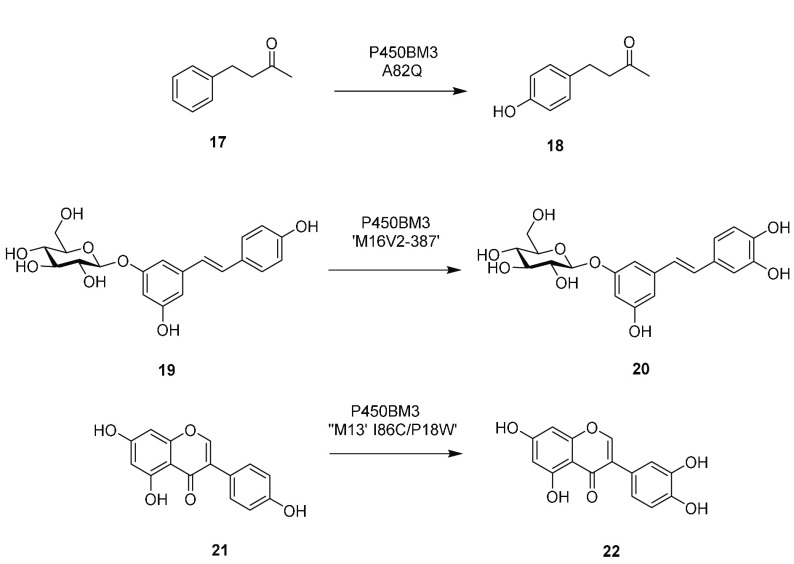
Hydroxylations of Further Aromatic Compounds by P450BM3 Mutants

Rousseau and co-workers assessed P450BM3 libraries for the hydroxylation of 4-phenylbutan-2-one **17** to give raspberry ketone (4-(4-hydroxyphenyl)-2-butanone) **18** (Scheme 5).^34^ Mutants which performed well in an indigo oxidation screen showed good correlation with improved hydroxylation activity, and mutant A82Q gave 90 μM min^–**1**^**18** from reactions containing 5 mM [substrate] at the 50 mL scale. A further screen of P450BM3 mutants by Le and co-workers revealed variant M16 V2-387, which catalyzed selective C3′ hydroxylation of the stilbene polydatin **19** to form the catechol (*E*)-astringin **20**, with 39-fold improvement over the parent M16 V2 variant.^35^ P450BM3 mutant “M13” was mutated to “M13” I86C/P18W, which displayed improved regioselectivity in the hydroxylation of the isoflavone genistein **21** to 3′hydroxygenistein **22** over its 8-isomer in a ratio of 12:1 compared to 1:1 for the wt.^36^

### Miscellaneous Aliphatic Hydroxylations by P450BM3 Variants

**Scheme 6:**
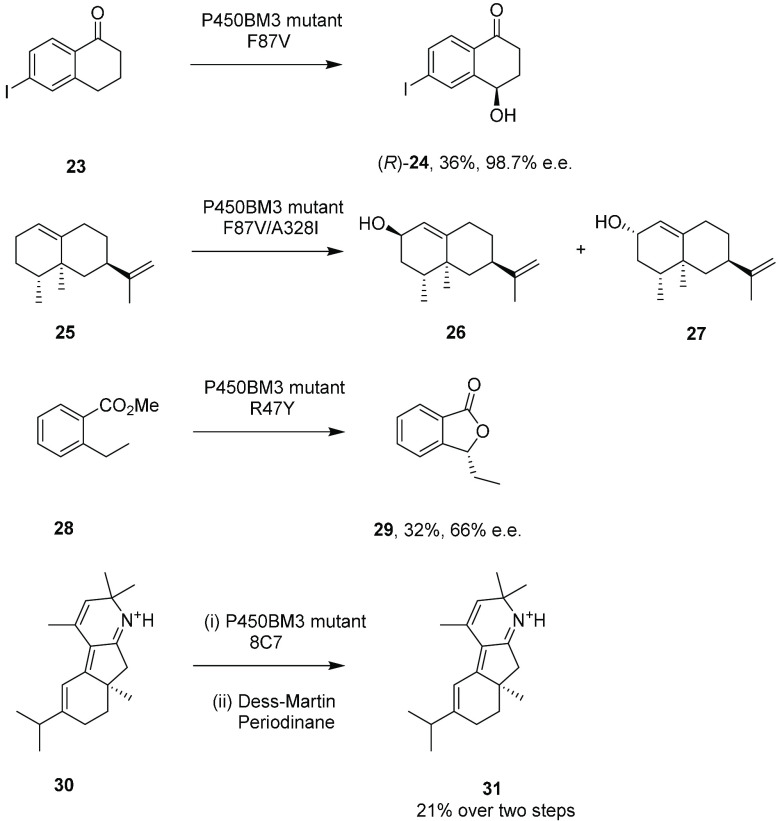
Miscellaneous Aliphatic Hydroxylations by P450BM3 Mutants

Reetz and co-workers exploited large P450BM3 mutant libraries to create a catalyst for the selective hydroxylation of 6-iodotetralone **23**, focusing on the hotspot F87 position.^37^ Mutant F87V was 99% regioselective and gave the (*R*)-product **24** with 36% yield and 98.7% ee at the 108 mg scale (Scheme 6).

Schulz and co-workers used mutant F87V/A328I as the first catalyst in a two-step synthesis of nootkatone, in which (+)-valencene **25** was transformed to mixtures of *cis-***26** and *trans*-nootkatol **27**, achieving 221 mg L^–1^ of the final product at the 20 mL scale.^38^ Pietruszka and co-workers screened 65 P450BM3 variants for the enantioselective allylic hydroxylation of ethyl-6-heptenoate.^39^ Mutant A74G/L188Q transformed the substrate to the (*S*)-alcohol with 90% selectivity over the terminal epoxide and with 95% ee, enantioselectivity that was also conserved for longer substrates from ethyl-7-octenoate to ethyl-10-undecanoate. Isolated yields of between 23 and 45% were obtained at the 60 mL scale employing 0.6 mmol substrate and pure enzyme. Further studies by the group focused on the enantioselective benzylic hydroxylation of benzoic acid derivatives by P450BM3 variants in pursuit of phthalide and isocoumarin products.^40^ Methyl-2-ethylbenzoate **28** was converted to isocoumarin **29** with (*R*)- or (*S*)-selectivity using the R47Y or F87V/L188Q variants, respectively, with 79 and 70% conversion. The reaction with R47Y was scaled up to 2.20 mmol in 210 mL to give the (*R*)-product in 32% isolated yield and 66% ee.

**Scheme 7:**
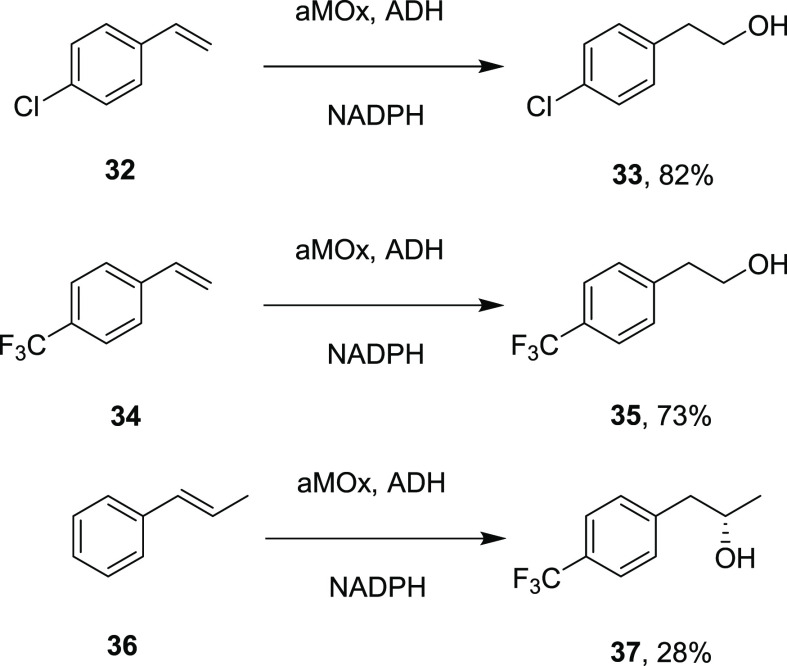
“Redox Hydration” of Alkenes by the aMOx Variant of P450_LA1_ Coupled with an Alcohol Dehydrogenase (ADH)^42^

Arnold and co-workers applied BM3 variant “8C7” to the selective oxidation of molecule **30** to give hydroxylated intermediates that are the direct precursors to the alkaloid nigelladine A **31**.^41^ A lysate of *E. coli* was used for a 160 mg scale oxidation of **30**, the products of which were subsequently oxidized to **31** using Dess–Martin periodinane in 21% yield after two steps. Arnold also reported the engineering of another redox self-sufficient P450, P450_LA1_ from *Labrenzia aggregata*, for the oxidation of styrene.^42^ Ten rounds of directed evolution were successful in changing the selectivity of P450_LA1_ from a 45:55 ratio of styrene oxide/phenacetaldehyde to 19:81 for the anti-Markovnikov product. This “aMOx” variant was coupled with an alcohol dehydrogenase for the “redox hydration” of alkenes **32**, **34**, and **36** at the 40–60 mg scale to alcohols **33**, **35**, and **37**, respectively, in the last case with an er of 99:1 (Scheme 7).

### Larger-Scale Hydroxylations by P450BM3 Variants

**Figure 2:**
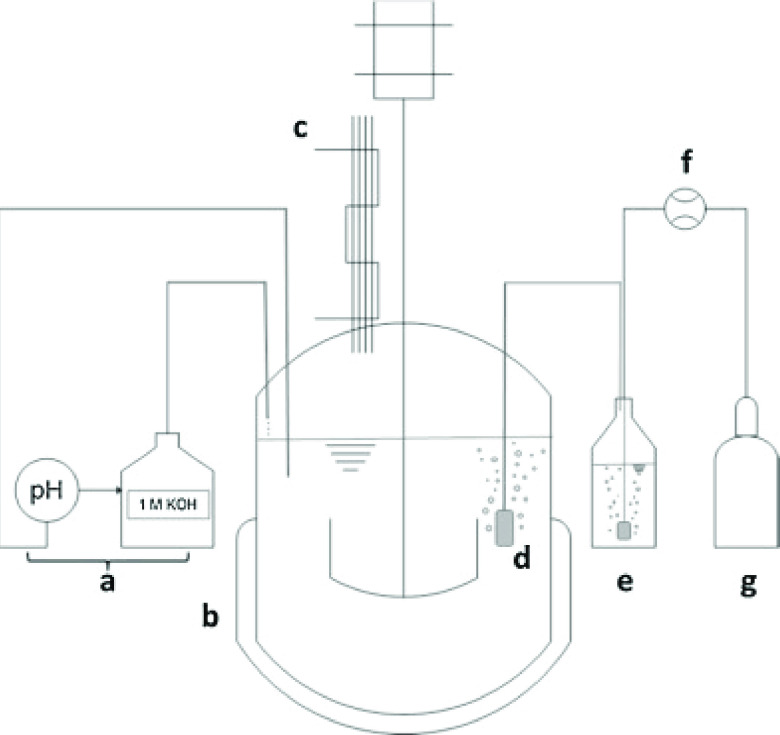
Reactor for α-ionone hydroxylation by the R47L/F87V/L188Q P450BM3 mutant. (a) Automatic titration device consisting of a pH meter and base titrator; (b) stirred tank batch reactor with a heating jacket; (c) reflux condenser; (d) bubble aeration via sintered frit; (e) gas washing bottle filled with water; (f) mass flow controller; (g) pressurized air connection. Reproduced with permission from ref (43). 2016, Elsevier.

The experiments described above show that screening was successful at identifying variants with altered selectivities, but oxygenations using in vitro P450BM3 rarely yielded products above the tens to hundreds of milligrams scale. However, if process factors, including substrate concentration and oxygen delivery, are considered together, gram-scale processes are achievable. Liese and co-workers used CFE containing mutant R47L/F87V/L188Q, coupled with glucose dehydrogenase (GDH) for NADPH recycling, to construct a scalable in vitro system for the hydroxylation of α-ionone.^43^ In studying factors such as the addition of cosolvents and detergents, oxygen supply, and the amount of enzyme, 1 L scale transformations of the substrate were achieved with a productivity of 4100 mol product/mol enzyme. The enzyme was applied in a bubble aerated stirred tank reactor (Figure 2) at 15 mM [substrate] with 5% methanol and an air-flow rate of 100 mL min^–1^ to give 97% conversion.

Nidetzky and co-workers established a gram-scale ω-2 hydroxylation of dodecanoic acid by wt P450BM3 and GDH using either soluble enzymes or coimmobilization onto Relisorb SP400 beads.^44^ P450BM3 was not stable on the carrier, so soluble enzymes were selected for application in a 500 mL reaction supplied with 80 mM (16 g L^–1^) substrate in 10% DMSO, using a feeding strategy based on oxygen feedback control. A TTN of 40 000 was achieved, giving a space-time yield (STY) of 0.6 g L^–1^ h^–1^ and 7.1 g of isolated product in 92% purity.

### Whole-Cell Bacterial Systems Expressing P450s

The limitations of applying in vitro preparations of P450s for scalable oxygenations has dictated that, with notable exceptions,^43,44^ whole-cell systems have been the major focus of study for larger reactions. Many of these again use either wt or variants of P450BM3.

**Scheme 8:**
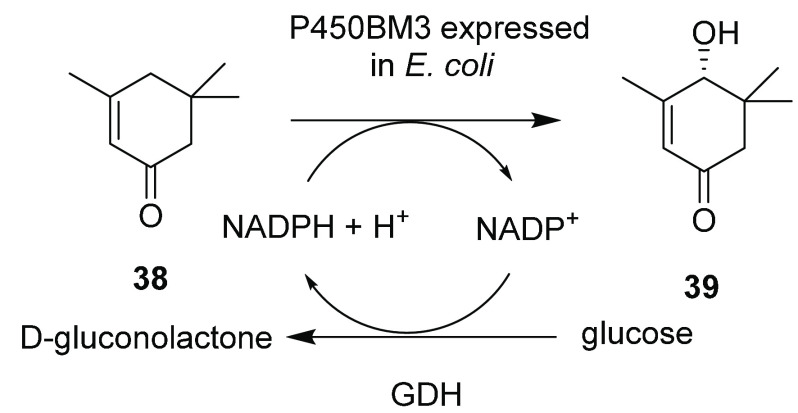
Hydroxylation of Isophorone by *E. coli* Expressing wt-P450BM3 GDH = glucose dehydrogenase.

The best recent example of this was presented by DSM, who produced the highest yielding P450BM3-catalyzed hydroxylation using a recombinant system.^45^ Freeze-fractured cells of *E. coli* expressing wt P450BM3 and a GDH for NADPH-recycling were employed for the regio- and enantioselective hydroxylation of isophorone **38** to 4-hydroxy α-isophorone **39** (Scheme 8) at the 100 L scale, permitting access to a hundreds of grams yield of product. Key to the success of this approach was screening reaction conditions at both the 20 mL and 1 L scales with an oxygen flow of 0.6 L h^–1^, in order to maximize oxygen availability. The largest transformations were performed at the 100 L scale in a 200 L fermentor, using cells derived from a 1000 L fermentation, with a stirrer speed of 140 rpm and an oxygen flow of 72 L h^–1^. Substrate at 6 g L^–1^ was added, and a productivity of 1 g L^–1^ h^–1^ was achieved, giving conversions of 80 and 82% and yields of the product of 51% and 61% with 99% ee, respectively.

### Bacterial Whole-Cell Hydroxylations of Steroids

**Scheme 9:**
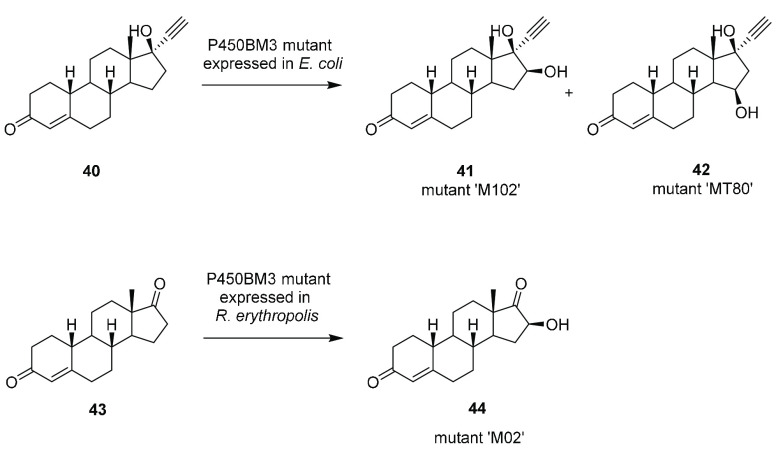
Hydroxylations of Norethisterone **40** and Norandrostenedione **43** by P450BM3 Mutants Expressed in *E. coli* and *R. erythropolis*

Reinen and co-workers screened P450BM3 libraries in vitro for the hydroxylation of the contraceptive norethisterone (NET) **40** (Scheme 9).^46^ Mutants “MT80” and “MT102” catalyzed regioselective transformations to 16-β-hydroxNET **41** and 15β-hydroxyNET **42**, respectively. Using whole cells of *E. coli* in a 1 L fed-batch system and with carbogen (80% oxygen, 20% carbon dioxide) as the oxygen supply, variant MT80 gave 47.6 mg L^–1^ of the 16β product **41**, whereas M102 gave 308.7 mg L^–1^ of the 15β product **42**, each with 99% regioselectivity.

Further transformations of steroids using P450BM3 variants employed *Rhodococcus erythropolis* as the host, as steroid uptake was thought to be superior in this strain.^47^ Following the identification of the P450BM3 mutant “MO2” for the conversion of norandrostenedione **43** to its 16β-hydroxylated derivative **44**, this was expressed in *R. erythropolis*, and the recombinant strain produced 0.35 g L^–1^**44** from 1 g L^–1^**43** at the 25 mL scale.

### Bacterial Whole-Cell Aromatic Hydroxylations

**Scheme 10:**
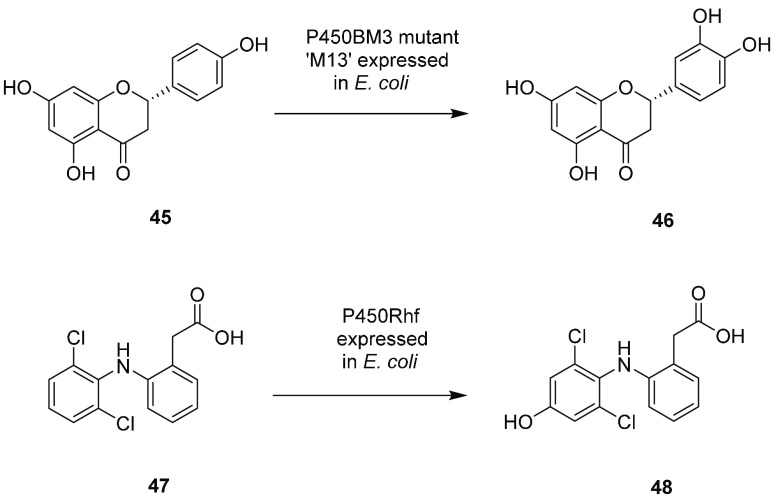
Aromatic Hydroxylations by Whole Cells of *E. coli* Expressing P450s

Chu and co-workers used P450BM3 mutants expressed in *E. coli* for the biotransformation of the flavonoid naringenin **45** (Scheme 10).^48^ Mutant “M13” catalyzed the 3′-hydroxylation of **45** at 100 μM concentration at the 3 L scale to give eriodictyol **46** with 50% conversion and 13.5 mg L^–1^ yield.

P450Rhf is a redox self-sufficient P450 of different structural organization to BM3, but which retains the advantages of a fused reductase-heme domain structure. Whole cells of *E. coli* expressing P450Rhf were applied to the 5′-hydroxylation of the anti-inflammatory compound diclofenac **47** (Scheme 10) in a fermentor at the 1 L scale,^49^ using a substrate concentration of 0.3 g L^–1^, with further additions of 0.1 g L^–1^ at 8 and 23 h. A product concentration of 0.36 g L^–1^ was achieved after 30.5 h at a rate of 11.7 mg L^–1^ h^–1^ and gave yields of 220–240 mg.

### Miscellaneous Aliphatic Hydroxylations by Bacterial Whole Cells

**Scheme 11:**
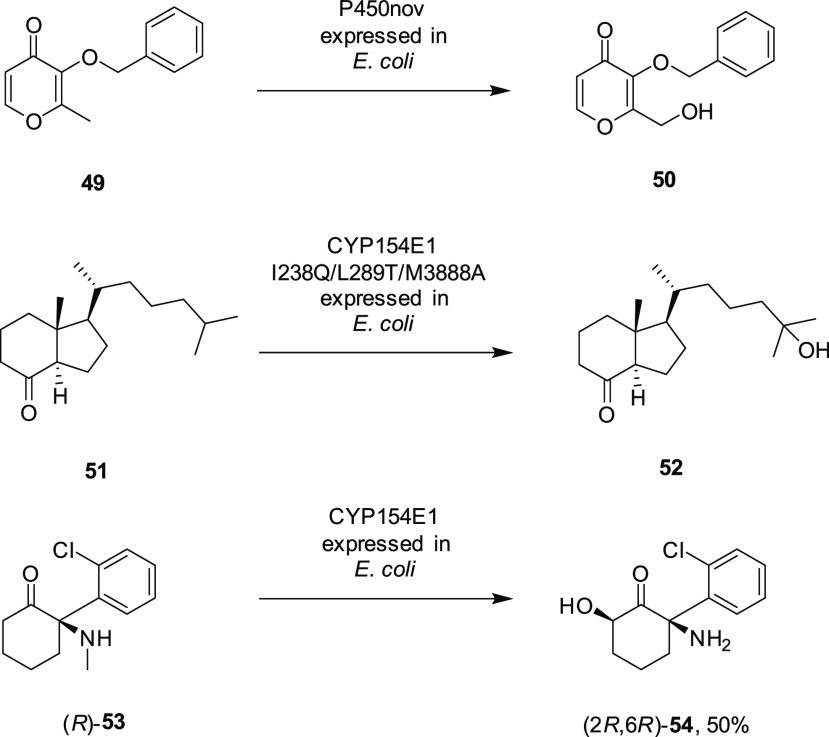
Miscellaneous Aliphatic Hydroxylations by Class I P450s Expressed in *E. coli*

The use of whole cells facilitates the deployment of “Class I” bacterial P450s that require FdR, Fd, and the heme domain for activity, through coexpression of these proteins in the whole-cell host. Ogawa and co-workers constructed a strain of *E. coli* expressing the Class I P450nov and cognate Fd and FdR from *Novosphingobium* sp. SB32149 for the conversion of the metal chelating compound **49** (BMAL) to 3-benzyloxy-2-hydroxymethyl-4-pyrone **50** (BMAL–OH) (Scheme 11).^50^

Two rounds of directed evolution gave a four-point mutant that was used in a 100 mL scale reaction, in which 5.2 g L^–1^ of the product **50** was produced from 8 g L^–1^**49** in a 250 mL fermentor. In a similar fashion, CYP154E1 from *Thermobifida fusca* was expressed in *E. coli* with PdX and PdR to create a system for the hydroxylation of Grundmann’s ketone **51** to its 25-hydroxy derivative **52**.^51^ In 5 mL scale reactions, a concentration of 2 mM substrate yielded 1.1 mM product after 24 h. CYP154E1 was álso engineered to oxidize (*R*)-ketamine **53** to the antidepressant (2*R*,6*R*)-hydroxynorketamine **54** via two-step *N-*demethylation and hydroxylation.^52^ Mutagenesis yielded variant I238Q/L289T/M388A, which, when expressed in *E. coli*, was used at the 10 mL scale at 5 mM [substrate] to give 12 mg product with 50% yield. The Class I enzyme P450pyr from *Sphingomonas* sp. HXN-200 was engineered by Li and co-workers to create mutants for the subterminal oxygenation of *n*-octane.^53^ One variant, A77Q/I83F/N100S/F403I/T186I/L302 V (P450pyrSM1), expressed in *E. coli*, hydroxylated octane to (*S*)-octanol with 99% selectivity and 98% ee at 5 mM concentration at the 10 mL scale. A further variant, I83F/N100S/T186I/L251 V/L302 V/F403I (P450pyrSM2) hydroxylated propylbenzene to (*S*)-1-phenyl-2-propanol with 98% selectivity and 95% ee on the same scale. A double mutant of P450pyr, I83M/I82T, identified from a screen of mutants targeting 22 residues, hydroxylated butanol to butanediol, again at 5 mM substrate and on a 10 mL scale.^54^

### Bacterial Whole-Cell Hydroxylations Using CYP153 Enzymes

**Figure 3:**
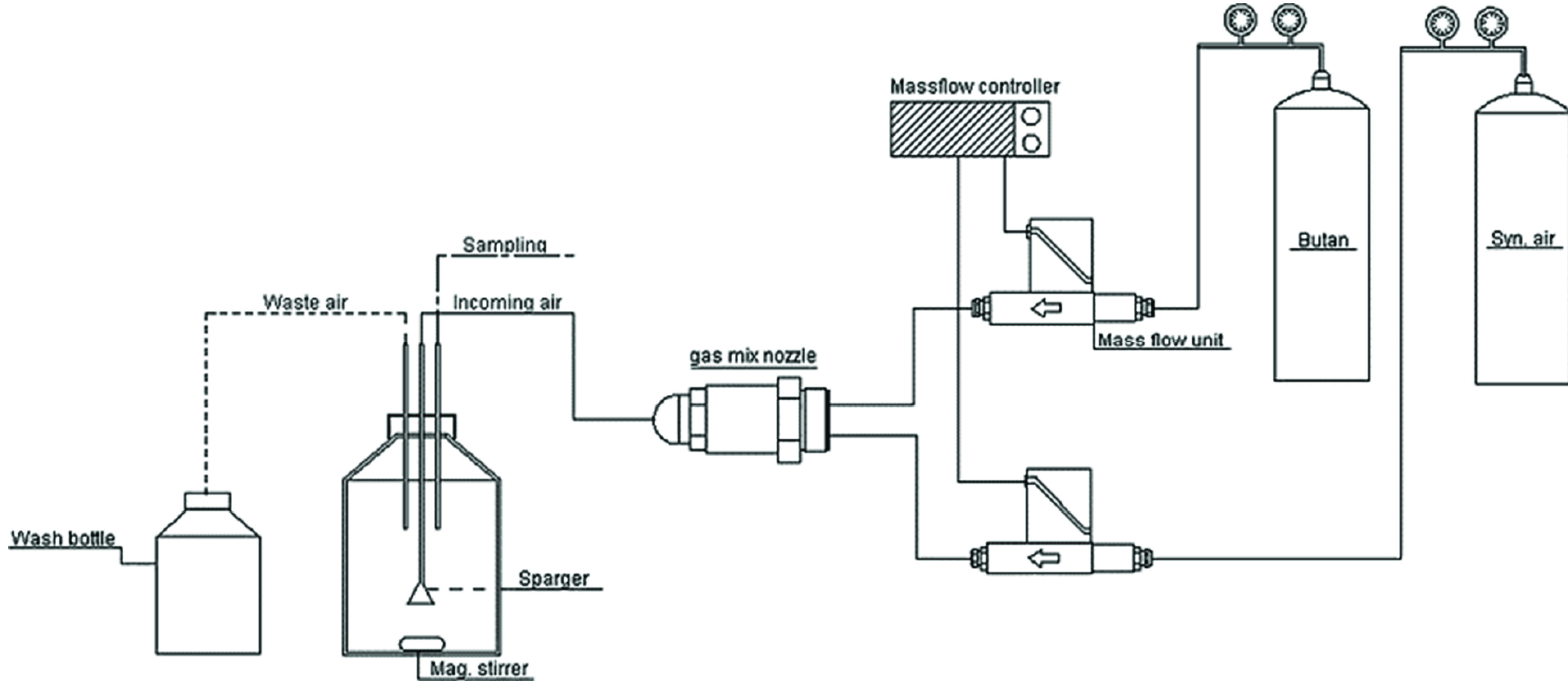
Schematic illustration of the apparatus used for the hydroxylation of gaseous butane by G254A mutant heme domain of CYP153A from *Polaromonas* sp. JS666. Reproduced with permission from ref (55). 2014, Elsevier.

Class I P450s of the CYP153 family have been the focus of several studies, as they have potential for the hydroxylation of alkanes and fatty acids. A variety of solutions to their electron transport requirements and cofactor provision have been explored. Hauer and co-workers fused the P450BM3 reductase domain to the G254A mutant heme domain of CYP153A from *Polaromonas* sp. JS666 (CYP153AP.sp.), which displayed activity for the hydroxylation of butane to 1-butanol.^55^ Cells of *E. coli* expressing the fusion protein oxidized gaseous butane, in a pressurized reactor at 15 bar, used to increase butane solubility (Figure 3). Using 61.2 mM butane, 4.35 g L^–1^ 1-butanol was produced with 96% selectivity. Hauer also employed a fusion of the CYP153 heme domain from *Marinobacter aquaeolei* with the P450BM3 reductase in a whole-cell system for the ω-hydroxylation of dodecanoic acid.^56^ Process limitations included substrate and product inhibition, biocatalyst stability, and cofactor supply. Introduction of the substrate in the solid form helped to address issues of substrate solubility, and in a 1 L bioreactor >5 mM product was obtained from 10 mM substrate.

A CYP153 homologue from *Polaromonas* sp. JS666 was coexpressed in *Pseudomonas putida* KT2440, with Fd, FdR and a hydrogenase that converts NAD^+^ to NADH at the expense of hydrogen.^57^ Cells of *P. putida* were challenged with 15% octane and produced 101 mg L^–1^ 1-octanol, although octanal and octanoic acid were also produced. In an alternative approach to cofactor provision, Harrison and co-workers coexpressed CYP153A6 with Fd, FdR, and a glycerol dehydrogenase in *E. coli* and evaluated the system for the hydroxylation of octane to 1-octanol.^58^ The use of mechanically permeabilized cells expressing all enzymes gave a catalyst with which a product titer of 61 mM was achieved, attributed to improved mass transport in the absence of the cell membrane. Substrate transport through the cell membrane was also addressed by Park and Choi, who coexpressed CYP153A from *M. aquaeolei* with an Fd and FdR but also FadL, a long chain fatty acid transporter, in *E. coli* deletion strain BW25113, in which beta-oxidation is blocked.^59^ The whole-cell system was then challenged with dodecane. Coexpression of the FadL protein increased the concentration of the product α,ω-dodecanediol from 0.12 to 0.69 mM, giving 178 mg L^–1^ from 20 mM dodecanol at a rate of 56 mg L^–1^ h^–1^. Effective substrate transport was also targeted by Lee and co-workers, who transferred the CYP153A operon from *M. aquaeolei*, which included genes encoding the cognate Fd and FdR, to *E. coli*, in combination with the AlkL alkane transporter from *P. putida.*(60) In a 5 L bioreactor containing 2 L of cell culture, and following addition of 200 mL of either dodecane or tetradecane, 1.5 and 2 g L^–1^ 1-dodecanol and 1-tetradecanol, respectively, were produced in 20 h with a dissolved oxygen level of 40%.

### Bacterial Whole Cell Hydroxylation of Cyclohexane

Buhler and co-workers have explored the application of a P450 from *Acidovorax* sp. CHX100 and its cognate Fd and FdR in the oxygenation of cyclohexane to cyclohexanol. In one study, the three proteins were coexpressed in *Pseudomonas taiwanensis* VLB120.^61^ A resting cell activity of 20 U g^–1^ cell dry weight (CDW) was recorded for the strain when challenged with 100 mM cyclohexane. The substrate was found to permeabilize the cells, reducing activity, but introducing it through the gas phase improved biocatalyst stability. Biocatalyst performance was improved by strain engineering,^62^ through altering the ribosome binding site (RBS) for the P450 gene, changing the plasmid copy number, and introducing a terminator to reduce “leaky” expression. This enhanced P450 expression improved the activity from 20 to 55 U g^–1^ CDW, permitting a yield of 82.5% from 5 mM cyclohexane. In the interest of creating a light-driven system for the cyclohexane oxidation, the same group cloned and expressed *Acidovorax* P450, Fd, and FdR in the photosynthetic cyanobacterium *Synechocystis* sp. PCC6803.^63^ In this system, both oxygen and the reduced cofactors would be provided by photosynthetic water oxidation. Small-scale reactions at 5 mM [cyclohexane] resulted in specific activities of 26 U g^–1^ CDW at 150 μmol photons m^–2^ s^–1^. Efficiency was improved using a two-phase system in which cyclohexane was supplied as a 20% solution in diisononyl phthalate (DINP), resulting in a maximum specific activity of 39 U g^–1^ CDW. Cyclohexane oxidation was then performed in a stirred-tank photobioreactor at the 1.2 L scale in a nonaerated two-phase system, giving a product yield of 2.6 g L^–1^.

### Whole-Cell Yeast and Fungal Systems Expressing P450s

While bacterial systems are attractive in terms of manipulatable genetics and growth rates, it is clear that some eukaryotic P450s, including human P450s, may benefit from expression in eukaryotic hosts such as yeasts and fungi, in which the natural P450 systems comprise a CPR and P450 domain.^64^ It is also true that the most successful scalable hydroxylation reactions have traditionally been carried out in whole cell yeasts or fungi, in many cases engineered using conventional strain improvement (CSI). The competition faced by recombinant P450 systems from native yeasts and fungi is illustrated by the biotransformation of decanoic acid methyl ester (DAME) by *Candida tropicalis*, reported by Schmid and co-workers.^65^ The first step in oxidation of DAME to dodecanedioic acid is a terminal hydroxylation by a native P450, CYP52A, and a CPR, each induced by the addition of substrate. Careful manipulation of the substrate feed rate and pH gave a yield of 66 g L^–1^ of the product on a 300 mL scale. Inspired by the catalytic performance of such strains, a range of approaches has been adopted toward the optimization of recombinant yeasts, with a view to emulating these productivities.

### Whole-Cell Hydroxylation Using Recombinant *Saccharomyces cerevisiae*

Recombinant expression in yeasts such as *S. cerevisiae* is a common strategy. For example, the steroid 14α-hydroxylase from *Cochliolobus lunatus*, P450lun, and its cognate CPR, were expressed in *S. cerevisiae,* creating a strain that catalyzed the 14α hydroxylation of androstenedione with 99% selectivity and 150 mg L^–1^ yield from a 250 mg L^–1^ substrate feed on a 20 mL scale.^66^ Yun and co-workers expressed two P450s from *Fusarium oxysporum*, *Fo*CYP539A7 and *Fo*CYP655C2 in *S. cerevisiae,* with CPRs from the host strain, but also *C. albicans* and the cognate CPR from *F. oxysporum.*(67) The recombinant strains catalyzed the ω-hydroxylation of C8–C12 saturated fatty acids, with yields of 36–58 mg L^–1^ but only when using a deletion strain of *S. cerevisiae* in which the acyl CoA oxidase gene Pox 1 was removed. The best strain contained the *Fo*P450s and the cognate CPR. The same group improved the performance of the strain expressing *Fo*P450s by coexpressing *Fo*CYP53A19 with *Fo*CPR and a GDH to improve cofactor supply for the P450 system.^68^ Cells of the recombinant strain converted 0.5 mM benzoic acid to 0.47 mM benzoic acid (94% conversion) with careful control of glucose concentration and pH.

**Scheme 12:**
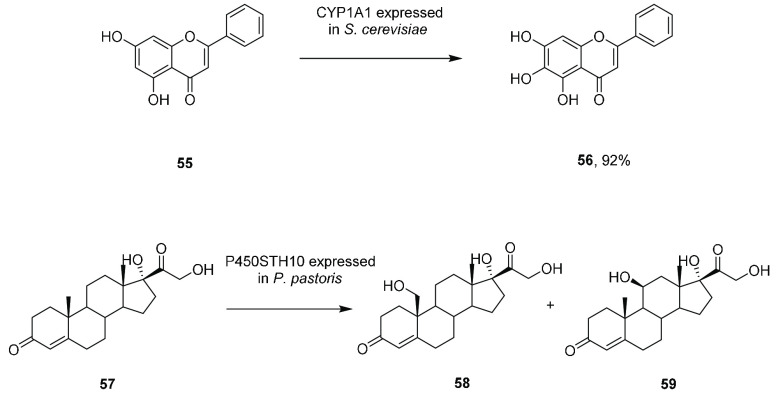
Hydroxylations by Recombinant Yeasts Expressing P450s

Chen and co-workers optimized promoter combinations for the expression of mutants of P450 *Sm*F3′H from *Silybum marinarum* and its cognate CPR in *S. cerevisiae*, to create a biocatalyst that catalyzed the 3′ hydroxylation of (2*S*)-naringenin to eriodictyol with yields of up to 3.3 g L^–1^ on a 2.5 L scale.^69^ The related flavonoid chrysin **55** (Scheme 12) was also hydroxylated by a recombinant strain of *S. cerevisiae*, in this case expressing human CYP1A1, to give the 6-hydroxylated product baicalein **56** at the 100 mg scale at 2 mM [substrate] with 92% conversion.^70^

To address the issue of insufficient cofactor supply to the P450 in an *S. cerevisiae* system, Zhang and co-workers developed a bioelectrocatalytic system in which electricity is used to recycle the reduced nicotinamide cofactor via external electron transfer, using neutral red (NR) as an electron shuttle.^71^ Using a strain in which CYP7B1 had been expressed, for the purpose of hydroxylating DHEA to 7α-OH-DHEA, 290 mg L^–1^ of product resulted from 400 mg L^–1^ substrate at the 140 mL scale, a productivity 2.4-fold greater than that achieved in the absence of NR.

### Whole-Cell Hydroxylation Using Recombinant *P. pastoris*

*P. pastoris* presents another option for heterologous expression of eukaryotic P450s as another genetically well characterized system used routinely for recombinant protein production. P450 STH10 and its cognate CPR from *Thanatephorus cucumuris* were identified as being able to hydroxylate steroids in the C-19 position. Coexpression of the genes in *P. pastoris* gave a strain that hydroxylated 1 mM 11-deoxycortisol **57** (Scheme 12) at the 500 mL scale, to give 19-hydroxy-11-deoxycortisol **58** and 11β-hydroxy-11-deoxycortisol **59**.^72^ Further P450s from this organism, CYP5150AP3 and CYP5150AN1, were also expressed with a CPR in *P. pastoris* and exhibited 7β- and 2β-hydroxylation activity toward **57** on the same scale.^73^*Pichia* is also a suitable host for human P450s such as CYP3A4. Glieder and co-workers performed the gram-scale hydroxylation of testosterone using cells of *P. pastoris* expressing CYP3A4 and human CPR.^74^ In 1.3 L of medium, 959 mg of substrate yielded 108 mg (11.3%) of 6β-hydroxytestosterone and 87 mg of 6β-hydroxyandrostenedione (9.1%) as the major products.

The construction of a chimeric fusion protein based on CYP105D7 permitted the creation of a *Pichia* strain competent for the 3′ hydroxylation of genistein to 3′-hydroxygenistein.^75^ The P450 was N-terminally fused to the transmembrane domain of CYP57B3 from *Aspergillus oryzae* and C-terminally fused to the CPR from *S. cerevisiae*. The strain converted substrate to product on a 2.5 L scale using 100 μM substrate, with a yield of 15 mg L^–1^.

Improvement of *Pichia* for hydroxylations has not been restricted to engineering of the P450 genes. Transcriptome analysis of *Pichia* engineered to express valencene synthase, *Hyoscyamus muticus* premnaspirodiene oxygenase (HPO), and a P450 reductase (PpHCV) revealed upregulation of a homologue of *Saccharomyces* DNA repair enzyme RAD52.^76^ In a strain in which RAD52 was overexpressed, the production of *trans*-nootkatol from valencene was elevated from a 20 to 40 mg L^–1^ cell culture at the 50 mL scale. When combined with optimization of organism cultivation, yields of 98 mg L^–1^ culture were obtained.

### Whole-Cell Hydroxylation Using Engineered Fungal Strains

**Scheme 13:**
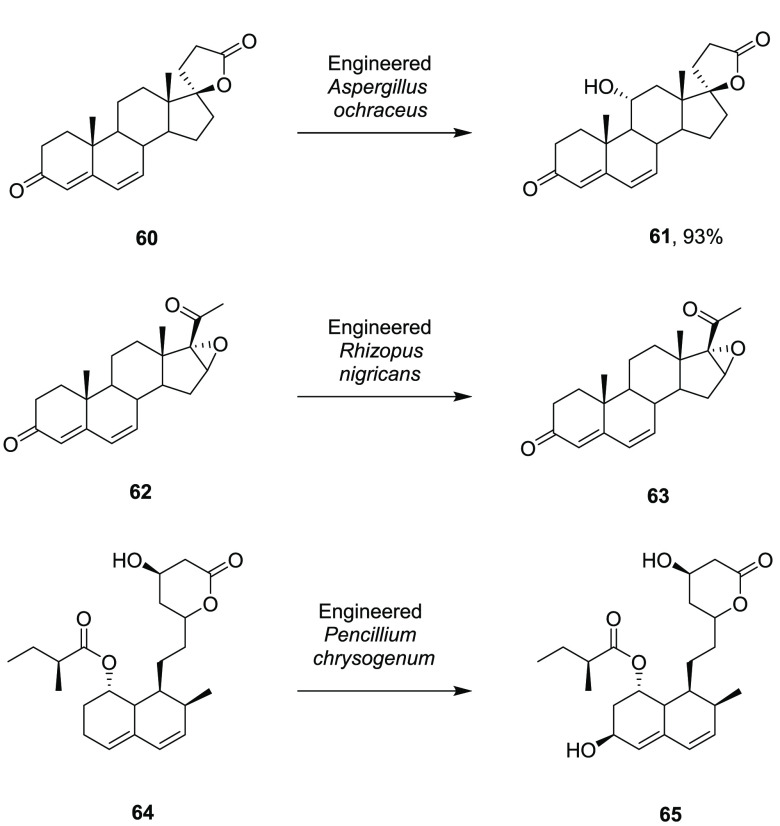
Hydroxylations by Engineered Fungal Strains

Given the history of scalable hydroxylations using fungi, it is unsurprising that contemporary methods have been applied to these organisms to alter or improve hydroxylation reactions. A strain of *Aspergillus ochraceus* M018, which catalyzed the 11α-hydroxylation of canrenone **60** (Scheme 13), was improved through homologous expression of the relevant P450, under the control of the Tr promoter, to create strain M010.^77^ Cells of the mutant strain, when challenged with 20 g L^–1^ of the substrate, gave 93% conversion to product **61** after 60 h compared to 75% after 96 h for the M018 strain. The hydroxylation of 16α,17-epoxyprogesterone (EP) **62** to 11α-hydroxy-16α,17-epoxyprogesterone (HEP) **63** by a native strain of *Rhizopus nigricans* ZXPO1 was enhanced by the heterologous expression within the strain of the glucose-6-phosphate dehydrogenase from *Rhizopus oryzae.*(78) Both biomass and biotransformation yields were increased, with a conversion of 38% after 52 h compared to 30% by the native strain, when challenged with 5 g L^–1^ EP at the 50 mL scale. Further attempts to increase cofactor provision were undertaken by Xu and co-workers, who increased productivity of 7α,15α-dihydroxydehydroepiandrosterone from DHEA by coaddition of the cofactor precursor nicotinic acid and 15 g L^–1^ glucose.^79^ This raised productivity by approximately 75% compared to controls. A fed-batch transformation with three sequential additions of 5 g L^–1^ of substrate gave 11.21 g L^–1^ of product after 60 h.

Protein and strain engineering were combined in a study of pravastatin production by *Pencillium chrysogenum.*(80) The compactin biosynthetic pathway from *Pencillium citrinum* was first introduced into *P. chrysogenum*, along with a gene encoding a fusion of P450 CYP105AS1 from *Amycolatopsis orientalis* with the reductase domain of P450 Rhf, with the objective of stereoselective hydroxylation of compactin. However, the P450 gave the opposite epimer, *epi*-pravasatin, to the one required, so enzyme engineering was performed to give mutant P450_prava_ that hydroxylated compactin **64** with the required stereoselectivity (Scheme 13). *P. chrysogenum* expressing both the compactin pathway and the P450_prava_ fusion protein were used in a 10 L fed-batch fermentor for the production of pravastatin **65** with yields of up to 6 g L^–1^.

## P450s Acting in a Peroxygenase Mode

Some of the complexities in the use of P450s can be addressed by exploiting their ability to act in a peroxygenase mode where, rather than generate Compound I through the delivery of electrons to the heme domain from NAD(P)H, the iron(IV) oxo species is generated directly using H_2_O_2_, exploiting what is known as the “shunt” pathway. Previous studies established that P450s, such as the heme domain of P450BM3, can be evolved for the improvement of peroxide driven biotransformations.^17^ More recently, using P450BM3, Nguyen and co-workers created a nine-point mutant for the 4’-hydroxylation of atorvastatin using only H_2_O_2_,^81^ although interestingly, the whole P450BM3 protein outperformed the heme domain in isolation. Further developments to the P450BM3 system for peroxide driven oxygenations employed a “dual function small molecule” (DFSM) approach,^82^ in which *N*-(w-imidazol-1-yl hexanoyl)-l-phenylalanine (Im-C6-Phe) is bound in the P450 active site so as to provide the imidazole side-chain as an acid–base catalyst, designed to mimic a homologous glutamate in UPOs, which use H_2_O_2_ as their natural route to Compound I. When Im-C6-Phe was included with mutant F87A/T268I/A184I in a biotransformation of propane, turnovers were 2-fold higher than had been recorded for UPOs with that substrate. This peroxygenase activity of P450BM3 was subsequently extended to the regioselective demethylation of methoxy-substituted aromatics using the DFSM technique;^83^ mutant F87A/T268I, in combination with Im-C5-Phe, gave a TON of 486 for the demethylation of anisole to phenol.

In addition to engineered peroxygenase activities, a family of P450s for which the natural mode of Compound I formation is through peroxide, has been described relatively recently.^18,19^ “P450 peroxygenases”, which include the enzyme OleT from *Jeotgalicoccus* sp. 8456,^84^ catalyze both the hydroxylation and decarboxylation of fatty acid substrates. The activity depends upon the formation of a salt-bridge between the carboxylic acid of the substrate and an arginine residue in the active site. This limits the substrate range of P450 peroxygenases, although native enzymes with the ability to enantioselectively oxidize styrenes to styrene oxides have been reported, including CYP119 from *Sulfolobus acidocaldarius*.^85^ These have been improved using site-directed mutagenesis, with ee’s of up to 91% reported for the epoxidation of *cis*-β-methylstyrene, although only on analytical scale.^86,87^ Despite these advances in the H_2_O_2_-driven peroxygenase activity of P450s, the use of peroxide to drive hydroxylations is best effected in Nature by unspecific peroxygenases (UPOs).

## Unspecific Peroxygenases (UPOs)

Unspecific peroxygenases (UPOs)^8,9^ are a class of secreted fungal hemoproteins that catalyze the oxygenation of organic substrates at the expense of H_2_O_2_ only and hence have no requirement for NAD(P)H or electron transfer proteins, in part addressing the “oxygen dilemma” (the dependence upon oxygen, balanced against the harmful ROS formation created during uncoupling processes) contextualized by Holtmann and Hollmann.^88^ This simplicity, coupled with high activity and stability, makes UPOs attractive alternatives to P450s for scalable oxygenations if systems could be facilitated for their heterologous expression and their activities engineered. Since their description by Hofrichter and co-workers,^20^ researchers have suggested that there are two major groupings of UPOs: “short” enzymes of approximately 29 kDa molecular weight, typified by *Mro*UPO from *Marasmius rotula*,^89^ and “long” enzymes of 44 kDa, typified by the *Aae*UPO from *Agrocybe aegerita*,^20^ the most studied UPO. While only a few UPOs from fungi have been identified and partially characterized, a larger study of their distribution^90^ in Nature revealed a large number of homologues, grouped in five subfamilies, the activities of which may provide valuable diversity.

### Oxygenations with wt UPOs

The activity of UPOs expressed from fungi, or in a recombinant *A. oryzae* system, had already revealed an interesting diversity of substrate specificity. Four peroxygenases displayed different selectivity toward the oxidation of 40 mM cyclohexane, with *Mro*UPO giving a 1.61:0.93 mM ratio of cyclohexanol to cyclohexanone. *Aae*UPO, by contrast, yielded a 3.05:0.05 mM ratio for these products.^91^ Wt *Aae*UPO was also reported to catalyze the oxygenation of 3.5 mM sulfides at the 3.5 mL scale.^92^ Thioanisole was converted to its (*R*)-sulfoxide with 95% conversion and 80% ee, although ee’s were improved for vinyl thiobenzene with values of >99%.

**Scheme 14:**
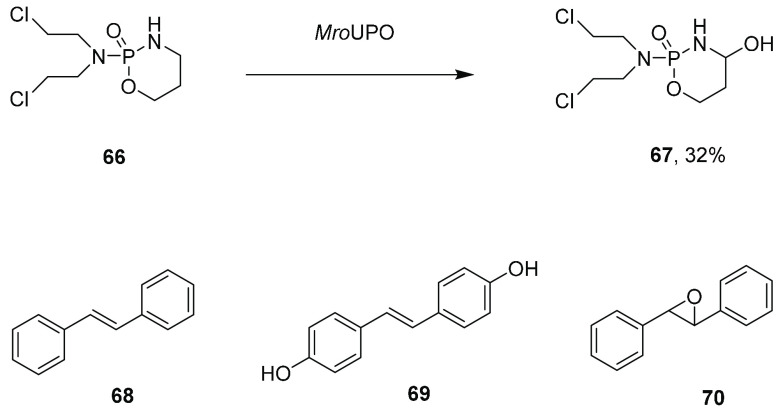
Some Oxygenations Catalyzed by Wt UPOs

The selectivity of *Mro*UPO for alkane hydroxylation was superior to that of *Aae*UPO, giving largely terminal hydroxylation of dodecane and tetradecane at a 0.3 mM concentration in 20% acetone to products with conversions of between 48% and 65%. *Aae*UPO gave primarily subterminally oxygenated products.^93^*Mro*UPO also catalyzed the chain-shortening of fatty acids through hydroxylation and oxidation in the α-position. In one example, *Mro*UPO, challenged with 0.1 mM decanoic acid at the 1–5 mL scale, gave nonanoic acid, although other products, including the α-hydroxyl-, α-keto, and diterminal acids, were also obtained.^94^ UPOs from *Marasmius* spp. were also found to catalyze side-chain removal from steroids such as cortisone^95^ through oxidation at C21 to form a *gem*-diol, followed by formation of a carboxylic acid. This underwent bond fission at C17 to yield adrenosterone as the major product with 70% yield from the 80 mg scale in 50 mL. *Aae*UPO was inactive toward this substrate, attributable to its narrower and longer heme access channel. In another steroid transformation, the UPO from *Chaetomium globosum* (*Cgl*UPO) catalyzed the oxygenation of testosterone to give both the 4,5-epoxide and 16α-testosterone in a 9:1 ratio, whereas neither *Aae*UPO nor *Mro*UPO converted the substrate.^96^ A 100 mg scale reaction in approximately 200 mL of buffer gave 65 and 7 mg isolated yields of the respective products. *Mro*UPO was also found to be the superior UPO among four including *Aae*UPO, *Cci*UPO, and *Cg*lUPO for the oxygenation of cyclophosphamide **66** (Scheme 14).^97^

At the 100 mL scale, 261 mg of **66** was transformed to 4-hydroxycyclophosphamide **67** in 32% yield when peroxide was delivered at 5 mM h^–1^, with only a small amount of the overoxidized 4-keto metabolite produced. These UPOs displayed very different selectivities for the oxygenation of *trans*-stilbene **68**, giving 100% conversion to the dihydroxylated 4,4′-dihydroxy stilbene **69**, except *Cgl*UPO which epoxidized the double bond to give product **70** with 100% selectivity.^98^*Cgl*UPO was also most selective for the epoxidation of fatty acids and fatty acid methyl esters, giving between 91 and 99% selectivity for the epoxidation of C14 to C20 *cis*-fatty acids, whereas other UPOs gave mixtures of epoxidized and hydroxylated products.^99^ Issues with selectivity were again observed with the transformation of 1,2-dihydronaphthalene by *Aae*UPO and *Cra*UPO.^100^ In analytical scale reactions, 2 mM substrate underwent epoxidation to yield the epoxide with 32% ee, but additional hydroxylation led to the naphthalene hydrates 2-hydroxy- and 1-hydroxy-1,2-dihydronaphthalene, which were subsequently aromatized, and then hydroxylated, to give naphthols. These examples illustrate the promise of UPO-catalyzed oxygenations, but also one of their limitations, which is the lack of selectivity when presented with some compounds with carbon atoms that are equally susceptible to oxygenation.

### Heterologous Expression of UPOs

One obstacle to scalable oxygenations using UPOs was their lack of availability, which in the research above depended upon their isolation from the culture medium of the relevant fungus. The development of heterologous systems for enzyme production therefore greatly facilitated their large-scale application, and also their engineering. An expression system using *Aspergillus niger*, which furnished enzymes for some of the studies described above, was patented by Novozymes in 2008.^101^ In 2014, Alcalde and co-workers reported the first expression of *Aae*UPO in both *S. cerevisiae* and *P. pastoris*(102) First, the heterologous expression of the *Aae*UPO gene enabled the construction of a directed evolution platform from which 9000 clones were assessed for improved expression and activity,^103^ resulting in a variant, PaDa-I, with nine point mutations from the wild-type sequence, which exhibited 1114-fold improved expression and 3250-fold improved activity. This variant contained four mutations in the signal sequence that promotes enzyme secretion, as well as five mutations within the enzyme itself: V57A/L67F/V75I/I248 V/F311L. Of these, structural studies showed that F311L enlarged the entrance to the heme channel from 8 to 12 Å, increasing substrate access to the heme.^104^

The gene encoding PaDa-I was transferred for expression in *P. pastoris* with an activity of 232 000 U L^–1^ or 217 mg mL^–1^, now constituting a robust platform for enzyme production.^103^*S. cerevisiae* continued to provide a platform for further directed evolution of *Aae*UPO. This included subjecting the PaDa-I gene to “neutral drift” experiments designed to elicit mutants with improved thermostabilities and tolerance to organic solvents, resulting in an improvement in *t*_1/2_ for one mutant of 34 min and improved tolerance to acetonitrile as a reaction cosolvent.^105^ Further experiments resulted in variants for which the peroxygenase/peroxidase activity ratio of *Aae*UPO was improved.^106^ Positions T120 and T320 were most significant in this case, with 4-fold increases in peroxygenase: peroxidase activity ratio obtained when mutations T120V and T320N(R) were coupled with mutation S226G, which had demonstrated improved thermostability over the PaDa-I variant.

**Scheme 15:**
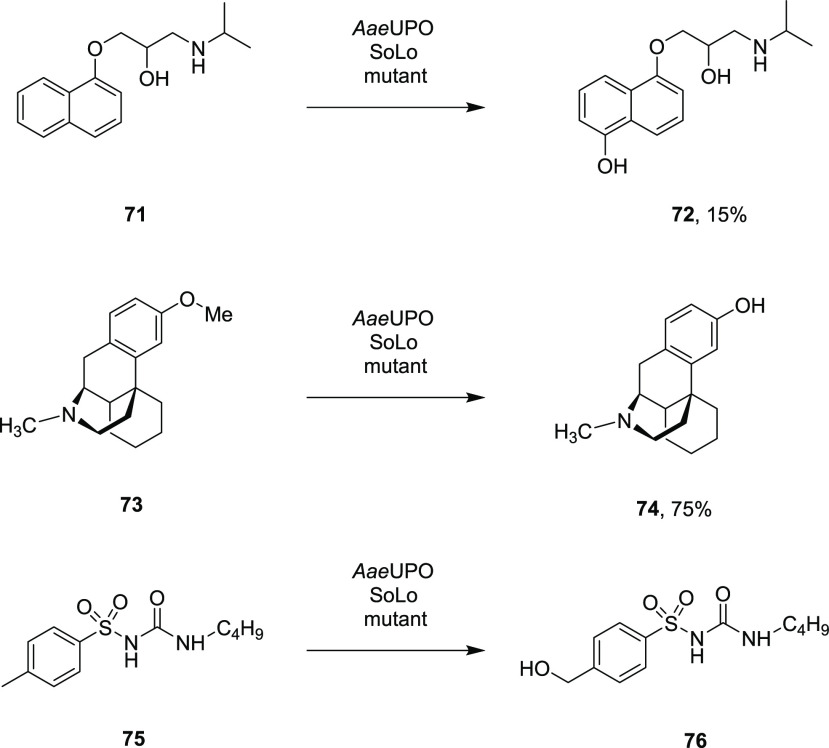
Oxidation of Pharmaceutical Molecules by Mutants of *Aae*UPO

These platforms for UPO evolution permitted further engineering of these enzymes. Another variant created from PaDa-I, with additional mutations G241D/R257K and named JaWa, displayed an improved ratio of peroxygenase/peroxidase activity^107^ and also an improved selectivity of 97% for the conversion of naphthalene to 1-naphthol over 2-naphthol compared to PaDa-I. Further improvements were obtained by mutating phenylalanines in the heme access channel to smaller residues; 12–16% increases in activity were obtained when F76 was mutated to A or L in combination with F191A.^108^ Structure-informed mutagenesis of JaWa permitted the creation of a further mutant with an F191S mutation, SoLo, giving a 15-fold improvement in the 99% regioselective transformation of propranolol **71** to 5′-hydroxypropanolol **72** with 445000 TTN (Scheme 15).^109^

In a 10 mL reaction, 50 mM propranolol was converted to **72** in 15.2% yield using *tert*-butyl hydroperoxide. Further drug metabolites were generated using the SoLo variant in a similar fashion.^110^ Dextromethorphan **73** was converted to dextrorphan **74** at the 136 mg scale in 100 mL to give a yield of 75.2%, with activity also recorded for the demethylation of naproxen and the hydroxylation of tolbutamide **75** to 4-hydroxymethyltolbutamide **76**, illustrating the utility of UPOs in the preparation of drug metabolites.

**Scheme 16:**
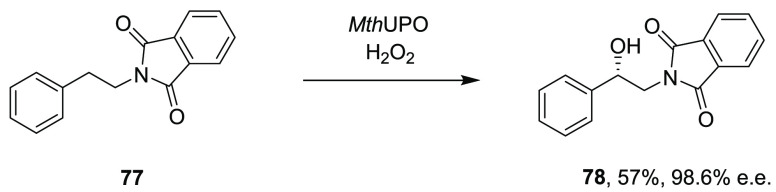
Hydroxylation of *N*-Phthaloyl-phenethylamine by UPO from *M. thermophila* (*Mth*UPO)^111^

Further work has enabled the rapid screening of expression constructs in *S. cerevisiae* for UPO production. Weissenborn and co-workers developed a modular tripartite system that permits the shuffling of 17 signal peptide sequences, UPO genes, and C-terminal tags encoding green fluorescent protein (GFP) variants for optimal expression and detection of target UPOs.^111^ The study revealed that optimal expression was sometimes obtained for UPOs with their noncognate signal peptide sequence. In this manner, UPOs from *Myceliophthora thermophila* (*Mth*UPO) and *Thielavia terrestris* (*Tte*UPO) were expressed and characterized, with *Mth*UPO catalyzing the epoxidation of styrene to give the (*S*)-epoxide product with 45% ee. Constructs were also transferred to *P. pastoris* for improved enzyme production. *Mth*UPO produced in this way hydroxylated *N*-phthaloyl-phenethylamine **77** (Scheme 16) to product **78** with 57% yield and 98.6% ee.

Failure to express in UPOs in an active form in *E. coli* had meant that evolution using this more accessible system was not possible. However, it was recently shown that some UPOs of the “short” variety can indeed be expressed in *E. coli*, expanding the possibilities of engineering these enzymes. Martínez and co-workers expressed *Mro*UPO in *E. coli* using a pET23a vector and altered its specificity toward oleic acid using in silico analysis coupled with mutation.^112^ Thus, wt *Mro*UPO, which produced a large proportion of epoxidized product, was converted to the I153F/S156 variant, for which the major products were oxygenated in the ω-1 position. This mutant was later applied to the 350 mL scale epoxidation of 0.1 mM α-linolenic acid to the 15(*R*)-, 16(*S*)-monoepoxide with 67% yield and 83% ee.^113^ Similarly, two further “short” UPOs, from *Collariella virescens* and *Daldinia caldariorum*, were also expressed in *E. coli*, illustrating the general applicability of the method.^114^ The *Collariella* UPO was mutated to an F88L variant, widening the heme access channel and giving improved selectivity for the oxygenation of linoleic acid, with reduced amounts of hydroxylated products.^115^

### Generating Hydrogen Peroxide for UPOs

**Scheme 17:**
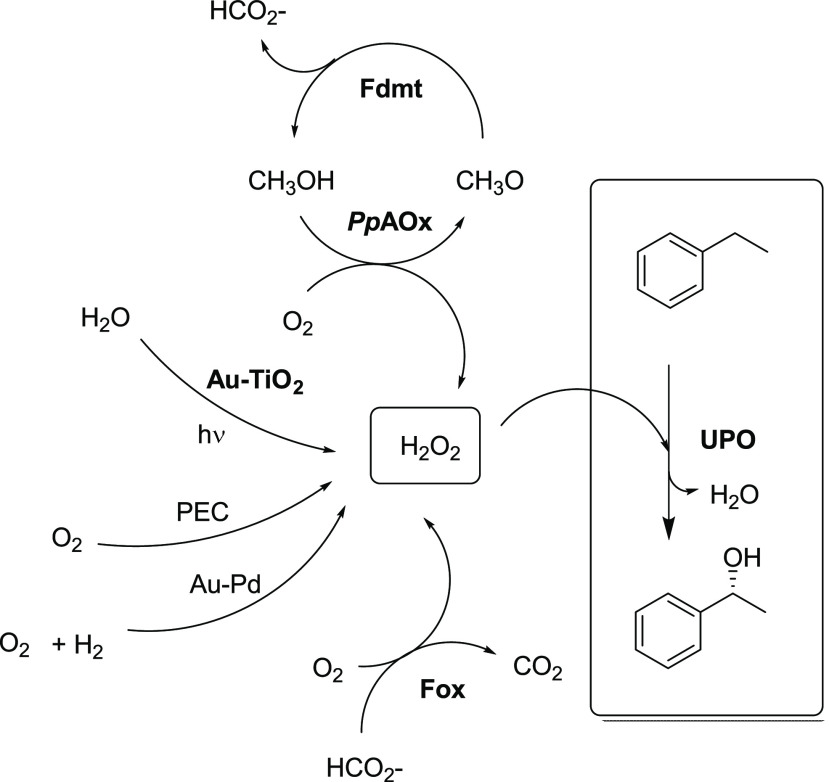
Selected Methods of Generating Hydrogen Peroxide for UPO-Catalyzed Oxygenations UPO = unspecific peroxygenase; Fox = formate oxidase; *Pp*Aox = *P. pastoris* alcohol oxidase; Fdmt = formaldehyde dismutase; PEC = photoelectrochemical.

With heterologously produced UPOs, rapid advances have now been made in their application and also in process engineering for scalable reactions. While many studies address new reactions, the question of how best to generate H_2_O_2_, in such a way as to not inactivate the UPO, has also been investigated (Scheme 17).

In many of the small-scale examples above, peroxide was delivered slowly either through a syringe pump or in aliquots, so as not to deactivate the UPO. To address the issue of peroxide sensitivity, Hollmann and co-workers showed that H_2_O_2_ could be generated in situ through the incorporation of an alcohol oxidase from *P. pastoris* (*Pp*AOx), which oxidized methanol to formaldehyde with concomitant generation of H_2_O_2_.^116^ Moreover, a formaldehyde dismutase (Fdmt) was incorporated into the reaction, catalyzing the transformation of formaldehyde to methanol and formic acid, increasing the turnover of methanol. In this way, ethylbenzene was converted to (*R*)-1-phenylethanol in 62% yield from a concentration of 50 mM. It also proved possible to use the oxidation of formate for peroxide generation through coupling a formate dehydrogenase with a flavin dependent reductase, Yqjm, which generates peroxide as a means of regenerating oxidized flavin in electron transfer reactions.^117^

This system was improved through the application of formate oxidase from *A. oryzae* (*Ao*Fox), which drove the production of H_2_O_2_ not only through the oxidation of formate but also from methanol, which served as the cosolvent in a selection of peroxygenase-catalyzed oxygenations.^118^ Hence, in the presence of 10% (v/v) methanol and *Ao*Fox, *Aae*UPO catalyzed the oxygenation of 100 mM ethylbenzene to (*R*)-1-phenylethanol with over 30% conversion in 144 h. In a further development, Alcalde and co-workers created a fusion of the *Aae*UPO SoLo variant with the flavin-dependent aryl alcohol oxidase (AAO) from *Pleurotus eryngii*, which generates peroxide as a byproduct when converting aryl alcohols to aldehydes.^119^ A construct “H”, in which the N-terminal UPO was connected to the C-terminal AAO by a linker of 110 amino acids, was applied to the biotransformation of dextromethorphan **73** shown in Scheme 15, but with the addition of 4-fluorobenzyl alcohol as the AAO substrate for peroxide generation. The fusion displayed a 2-fold increase in production of dextrorphan **74** after 15 min of incubation, compared to a mixture of the individual enzymes, suggesting improved substrate channelling within the fusion.

One way to address the peroxide supply without recourse to additional enzymes is to exploit photocatalytic oxidation reactions for the oxidation of water to H_2_O_2_.^120^ Gold-containing titanium dioxide, when irradiated with visible light, catalyzed the oxidation of water to H_2_O_2_, which was then used by *Aae*UPO to hydroxylate ethylbenzene. To minimize enzyme inactivation, *Aae*UPO was immobilized on a poly(methyl methacrylate) resin and used in combination with hydrophobic rutile Au-TiO_2_. This gave a robust catalytic system with total turnovers increased from 2000 to 21 000 over reaction times of up to 120 h. A range of alkanes and cycloalkanes were converted to mixtures of alcohols and ketones including a semipreparative reaction in which 10 mM ethylbenzene was converted to the product with a 81% isolated yield and 97.4% ee. It was later determined that increases in light intensity and temperature increased productivity, but only up to the point at which these factors caused denaturation of the enzyme owing to increased generation of reactive oxygen species (ROS).^121^ Photocatalysis was also used for reductive activation of oxygen using electrons from water. A photoelectrochemical (PEC) tandem cell structure, consisting of an FeOO/BiVO4 photoanode, a CIGS solar cell, and a CN/rGO cathode, enabled first the oxidation of water at the anode, followed by oxygen reduction at the cathode, to give H_2_O_2_.^122^ This was coupled to the *Aae*UPO-catalyzed hydroxylation of ethylbezene again, giving a TTN of 43 300. A further system employed flavin molecules immobilized on single walled carbon nantotubes which, when irradiated with UV light, stimulated the production of H_2_O_2_ from oxygen, enabling *Aae*UPO-catalyzed hydroxylation of 100 mM ethylbenzene to 17 mM product over 50 h with a TTN of 123 000.^123^ Nitrogen-doped carbon nanodots (N-CNDs) were used as an alternative catalyst for photoactivated H_2_O_2_ generation.^124^ If coimmobilized with *Aae*UPO in alginate beads, this enabled the hydroxylation of cyclohexane as a neat substrate when N-CNDs and enzyme were applied in an 8:1 (mg/nmol) ratio, enabling a TTN of 7806 and a yield of 2.25 mM cyclohexanol over 96 h. Further transformations in neat media were achieved with *Aae*UPO immobilized on the polyacrylic support Immobead IB-COV-1, for the epoxidation of styrenes.^125^ At the 10 mL scale with a *tert*-butoxide delivery rate of 5 mM h^–1^, *cis*-β-methylstyrene was converted to 360 mM (1*R*,2*S*)-β-methylstyrene oxide and with a TTN of 8500. *Aae*UPO was also immobilized on PVA PEG gel beads and then applied to a 100 mL scale stirred tank reactor for the transformation of 5 mM diclofenac.^126^ In this way, 414 mg of 4′-hydroxy diclofenac was produced with a TTN of over 61 000. Fe-glass EziG matrices were also suitable supports for *Aae*UPO immobilization.^127^ Using *Aae*UPO with a C-terminal histidine tag, the enzyme was immobilized and applied to the hydroxylation of 10 mM ethyl benzoic acid with over 80% conversion, and could be reused for multiple cycles.

Peroxide was also generated from hydrogen and oxygen in water using AuPd catalysts at ambient pressure and at 40 bar, which has the advantage of no ROS formation as with photocatalysis.^128^ In 10 mL scale reactions using 10 mM cyclohexane with 80% hydrogen and 20% air introduced continuously at 2 bar and 2.5 wt % Au–2.5 wt % Pd/TiO_2_ as a catalyst, TTNs of 25 300 were achieved with an 87% yield of cyclohexanol in 16 h. Ethylbenzene at 20 mM was converted to 13.1 mM phenylethanol and 2.9 mM acetophenone in 16 h with a TTN of 51 400, and a TTN of 201 000 was achieved through sequential additions of 30 mM substrate to give 46 mM phenylethanol and 14 mM acetophenone in 64 h. The generation of H_2_O_2_ from oxygen was also addressed using an electroenzymatic approach, which offers a highly atom economical approach as it only requires oxygen, electrons, and protons. In a glass reactor fitted with a gas-diffusion electrode, H_2_O_2_ was continuously generated and supported the transformation of ethylbenzene with a TTN of 400 000 at a current density of −10 mA cm^–2^ and an STY of 13.2 g L^–1^ day^–1^.^129^

### Scaled-Up Biotransformations Using UPOs

**Figure 4:**
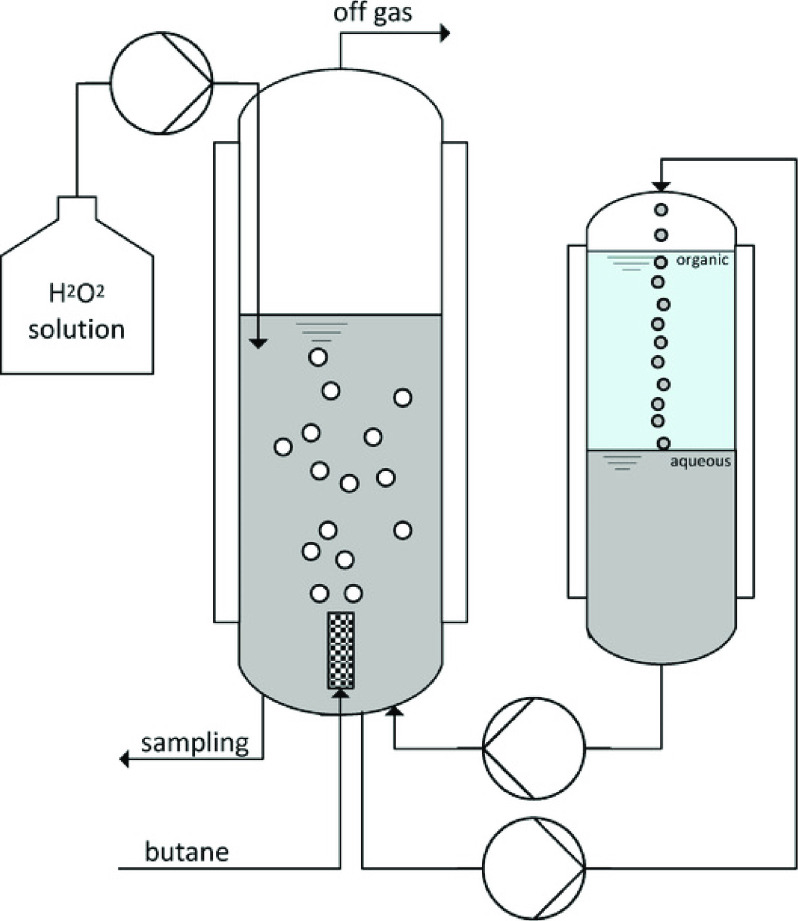
Experimental setup for butane oxygenation by *Aae*UPO in a bubble column reactor (middle) coupled with an extractive column (right). From ref (130). CC BY 4.0.

The potential of UPOs for scaled-up reactions was realized by Liese and co-workers, who performed a 2 L scale oxygenation of butane to butan-2-ol using *Aae*UPO.^130^ A bubble column reactor permitted the controlled introduction of both H_2_O_2_ and butane into the mixture at rates of 882 mM/136 mL h^–1^ and 61.4 L h^–1^ (Figure 4). Here, 8.5 g of 2-butanol and 5.6 g of butanone were recovered from the experiment after 4 h with overoxidation to butanone and biocatalyst stability being the major issues.

The scale-up of UPO reactions will be facilitated by the ability to produce the enzyme on a scale sufficient to furnish larger oxidations. Tonin and co-workers reported the production of *Aae*UPO at the 2480 L scale, which produced 170 g of the recovered enzyme.^131^ The large amount of UPO produced permitted its application to a 100 mL scale hydroxylation of ethybenzene in a two-liquid-phase system, giving a product concentration of 14.4 g L^–1^ in the organic layer after 7 days. The application of UPOs in such systems will benefit from evolution experiments targeted at increasing their stability to organic solvents. Alcalde and co-workers showed that a variant of the PaDa *Aae*UPO, WamPa, which features nine additional mutations, displayed 23-fold greater stability in the presence of 30% acetonitrile.^132^

## Summary

In this Perspective, we have surveyed recent advances in biocatalytic oxygenations by hemoproteins, with an emphasis on reactions that result in isolatable yields from the tens of milligrams to, in one case,^45^ kilogram quantities. The range of catalysts, from cell-free preparations, in soluble or immobilized form, to permeabilized and intact cells, has encouraged imaginative approaches to the optimization of each, with specific considerations applied to their distinct challenges.

It is clear that protein engineering will continue to play a vital role in the generation of new selectivities, but also in the creation of more active and stable enzymes. For P450s, the evolution methods have been facilitated through straightforward expression in *E. coli*. For UPOs, the examples are less well-distributed, owing to current requirements for expression in yeasts, but the development of these systems and also the encouraging work on the expression of active UPOs in *E. coli* mean that the engineering of UPOs in the mode of P450s should be more prevalent in the coming years.

The engineering of hemoproteins tailored for specific reactions provides the raw material for process development and scale-up. The requirement for both nicotinamide cofactors and cofactor recycling remains a process complication for the application of cell-free P450s, but examples in this survey^43,44^ show that consideration of factors that optimize substrate solubility and oxygen delivery can furnish processes that can deliver products on the gram to kilogram scale. However, it may be considered that productivities are still substantially lower than may be required, especially for the production of bulk chemicals. It is also encouraging that some of the larger scalable examples are not restricted to native substrates of P450BM3 but include structurally unrelated compounds such as α-isophorone.^45^ The process stability of P450s is a persistent challenge for cell-free reactions, but one that may conceivably be addressed by further protein engineering or enzyme immobilization approaches. The expense of in vitro P450 processes, which is not often quantified in academic papers, must also be considered against the possibilities offered by whole-cell reactions that do not require the supply of exogenous cofactor.

The complications of cell-free processes make whole-cell P450 reactions appear attractive; therefore, their simplicity, tolerance to higher concentrations of substrate, and consequent superior productivities at scale^65^ suggest that these may be the catalysts of choice for scalable oxygenations. However, overmetabolism and side reactions with competing selectivity, particularly in wt strains, can depress yields and complicate downstream processing. Hence, the developments in the expression and application of P450s in recombinant host strains is very encouraging, although examples have shown that the productivities of non-native enzymes within recombinant strains can be reduced. These disadvantages can be addressed when a whole-organism approach to strain engineering is adopted,^69,76^ and techniques such as CRISPR-Cas9, which is already being used in yeasts^133^ and fungi^134^ for biotechnological applications including those related to P450,^135^ should accelerate the development of improved strains.

Given the process complications of P450s in vitro and the complexities of strain engineering in the optimization of in vivo reactions, UPOs present themselves as a simple and applicable alternative for scalable reactions, especially in the chemical laboratory. Despite their sensitivity to H_2_O_2_, the first scale-up reactions are showing that, when peroxide delivery is addressed, the enzyme can be deployed effectively for the gram-scale generation of oxygenated products.^130^ However, the examples in this survey show that the lack of selectivity, in the form of either overoxidation or oxygenation at multiple sites, is an ongoing issue. The increasing availability of new UPO sequences and the methods to engineer them suggest promising avenues for exploration in a bid to acquire better and more diverse selectivity.

In summary, given the advances in enzyme discovery, molecular biology, and process biochemistry, hemoprotein biocatalysts can still be considered to have considerable potential for selective and sustainable oxygenation reactions. However, the success of each reaction will depend strongly upon the selection of the right enzyme or host organism and the mode of oxygen or peroxide delivery. The stability of the biocatalysts in question under process conditions must also be addressed, and further work on enzyme engineering and biocatalyst formulation will also be important targets of research in the future.

## Abbreviations

P450: Cytochrome P450
UPO: unspecific peroxygenase
Fd: ferredoxin
FdR: ferredoxin reductase
CPR: cytochrome P450 reductase
*Pp*Aox: *Pichia pastoris* alcohol oxidase
Fdmt: formaldehyde dismutase
Fox: formate oxidase.
